# Potential of Skin Microbiome, Pro- and/or Pre-Biotics to Affect Local Cutaneous Responses to UV Exposure

**DOI:** 10.3390/nu12061795

**Published:** 2020-06-17

**Authors:** VijayKumar Patra, Irène Gallais Sérézal, Peter Wolf

**Affiliations:** 1 Center for Medical Research, Medical University of Graz, 8010 Graz, Austria; vijaykumar.patra@medunigraz.at; 2 Research Unit for Photodermatology, Department of Dermatology, Medical University of Graz, 8010 Graz, Austria; 3 Department of Medicine, Unit of Rheumatology, Karolinska Institutet, 171 77 Solna, Sweden; igallaisserezal@chu-besancon.fr; 4 Department of Dermatology, Besançon University Hospital, 25000 Besancon, France

**Keywords:** ultraviolet radiation, skin inflammation, skin microbiome, probiotics, prebiotics, photobiology, photoimmunology

## Abstract

The human skin hosts innumerable microorganisms and maintains homeostasis with the local immune system despite the challenges offered by environmental factors such as ultraviolet radiation (UVR). UVR causes cutaneous alterations such as acute (i.e., sunburn) and chronic inflammation, tanning, photoaging, skin cancer, and immune modulation. Phototherapy on the other hand is widely used to treat inflammatory skin diseases such as psoriasis, atopic dermatitis, polymorphic light eruption and graft-versus-host disease (GvHD), as well as neoplastic skin diseases such as cutaneous T cell lymphoma, among others. Previous work has addressed the use of pro- and pre-biotics to protect against UVR through anti-oxidative, anti-inflammatory, anti-aging, anti-carcinogenic and/or pro-and contra-melanogenic properties. Herein, we discuss and share perspectives of the potential benefits of novel treatment strategies using microbes and pro- and pre-biotics as modulators of the skin response to UVR, and how they could act both for protection against UVR-induced skin damage and as enhancers of the UVR-driven therapeutic effects on the skin.

## 1. Introduction

### 1.1. Skin Microbiome

The healthy human skin harbors multitude of diverse and complex communities of bacteria, fungi, viruses, archaea, and mites, collectively called the skin microbiome [1,2,3,4]. The skin displays striking variation of microbial composition across body sites, shaped by their physical, chemical, and biological features [5,6,7]. Unlike the nutrient rich intestinal environment, the skin surface lacks many nutrients beyond basic proteins and lipids. In order to survive in this hostile environment, the resident microbes have to adapt and utilize available resources present in the stratum corneum (sphingolipids, amino acids, peptides, nitric oxide), sweat (NaCl, H_2_O, HCO_3_^+^, glucose, amino acids, free fatty acids, urea, ammonia, lactate, vitamins, peptides, sterols, mucopolysaccharides) and sebum (free fatty acids, sterols, squalene, wax esters). In addition, the skin microbiome must maintain a constant interaction and healthy interplay with the skin’s immune system for its survival. Studies using germ-free animals have shown a crucial role of skin microbiome in physiological and immunological functions. For example, *Staphylococcus epidermidis*, one of the dominant skin-associated bacteria produces several antimicrobial compounds and proteases that can limit the formation of biofilms by pathogenic species. Colonizing the skin with *Staphylococcus epidermidis* remodels the skin immunity by inducing IL-17a+ CD8+ T cells that migrate to the epidermis, enhances the immunity and limits pathogen invasion [5,8]. Numerous studies suggest that the skin microbiome is intricately involved in a wide range of molecular and cellular processes within the skin and beyond [9,10]. Thus, the nutrients that play an important role in shaping the individual differences in microbial signature ultimately contribute to health and diseases.

### 1.2. Ultraviolet Radiation (UVR)

UVR, especially UV-A (315–400 nm) and UV-B (290–315 nm) is one of the most prominent external factors affecting the skin. UV-A rays are either UV-A1 (340–400 nm) or UV-A2 (320–340 nm). The penetration of UV-A through the skin layers (epidermis and dermis) is about 5–10 times higher than that of UV-B [11], and UV-A (like UV-B) is known to play a more major role in skin aging, wrinkling (photoaging) and takes part in initiating and promoting skin cancers. This carcinogenic effect is due to the action of UV-A on various endogenous photosensitizers to produce reactive oxygen species (ROS), which can damage both the DNA and its cellular repair machinery. In contrast to UV-A, UV-B can only penetrate through the epidermis and not the dermis. UV-B exposure is biologically more active by causing sunburn and other physiological changes in the skin, which includes the delayed and more long-lasting tanning. The intensity of UV-B radiation (unlike UV-A) vastly varies depending upon geographic location, time, and season. One of the prominent targets of UV-B is cellular DNA, which can absorb UV-B radiation leading to the formation of cyclobutane pyrimidine dimers [12], (6-4) photoproducts [13] and other lesions in the DNA. In addition, UV-B causes significant adverse effects on organisms such as bacteria [14,15], cyanobacteria, phytoplankton, algae [16], plants [17], animals and humans [18]. Some organisms have evolutionary developed UV-absorbing pigments as the first line of defense, though they cannot prevent UVR completely from reaching the DNA in superficial tissues [19]. DNA repair systems, such as nucleotide excision repair, superoxide dismutase (SOD), catalase (CAT) and peroxidase (POD), and vitamins, such as B, C, D and E, play a protective role against UV-B [19]. Vitamin D3 (cholecalciferol) is mainly generated in the skin as a response to UVR and is further converted to the principal biologically active form 1, 25 (OH)_2_D_3_ with the help of various enzymes. Vitamin D is one of the most important nutrients for optimal health and wellbeing. It is known to have a variety of potential benefits such as prevention of cancers and autoimmune diseases [20,21], reduction of high blood pressure [22], positive regulation of immune system [23] and antimicrobial activity [24,25,26]. UVR also causes systemic effects such as increasing blood concentrations of inflammatory (IL1β, TNF-α) and metabolic (low/high density lipoproteins) factors [27,28]. UVR impacts the skin-brain axis by releasing neuro-endocrine-immune factors such as β-endorphin and corticotropin-releasing hormones from the skin into the blood circulation. Then, these factors exert endocrine and opioidergic effects on the central nervous system [29]. Indeed, behavioral effects of therapies using UVR are described. Psoralen used together with UV-A (PUVA) treatment in early-stage mycosis fungoides seems to have a positive impact on patient’s quality of life and psychological wellbeing [30]. This opioidergic effect is thought be the result of UV-induced production of endorphins (which modulate itch, pain and reduce stress) by interacting with the neuroendocrine system [31,32,33,34]. Moreover, UVR is reported to induce the production of endocannabinoids in serum [35]. Together, those mediators may favor and support psychologic wellbeing. Furthermore, recent study shows a striking correlation between serum concentrations of 25(OH)D levels and abundance of various microbes in the gut, thereby suggesting an interplay between UVR exposure and the skin-gut axis [36]. Effects of UVR on the local and systemic levels have been very well reviewed elsewhere [29,37,38,39]. This article will focus on the potential of skin microbiome and pro- and prebiotics on local responses to UVR exposure.

## 2. Perspectives

### 2.1. UVR-induced Pigmentation and Antioxidation

**Figure 1:**
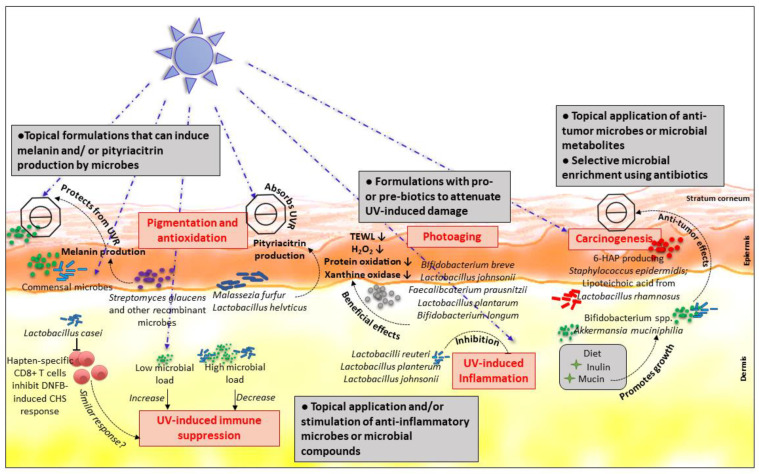
UV-induced effects on the skin and potential treatment strategies using microbes, pro- or pre-biotics. Topical formulations or application of recombinant microbes capable of inducing melanin and/or UV-absorbing compounds such as pityriacitrin could be used for pigmentation and antioxidation. Microbes or pro- or pre-biotics that can prevent and/or reduce UV-induced increase of transepidermal water loss (TEWL), hydrogen peroxide (H_2_O_2_) levels, oxidation of proteins and xanthine oxidase activity can have beneficial effects on photoaging. Novel strategies such as selective microbial enrichment using topical antibiotics, and/or application of anti-tumor, anti-inflammatory microbes or microbial metabolites/compounds could be beneficial to prevent UV-induced skin cancers and reduce UV-induced skin inflammation.

Skin pigmentation has long been believed to be the most important photoprotective factor, with melanin (especially eumelanin) functioning both as broadband UVR absorbent (of 50–70% of UVR) and possessing antioxidant and radical scavenging properties [40]. The underlying regulatory mechanisms leading to pigmentation are not well understood. Extensive data, however, suggests that UVR-induced DNA damage initiates signals that increases the melanogenesis. Melanin is produced in dendritic melanocytes (in specific ovoid organelles knowns as melanosomes), which account for mere 1% of the epidermal cells. Melanogenesis is regulated via various mechanisms such as transcriptional regulation, intracellular signal transduction pathways with cAMP responsive element being the critical factor [41] and reaction substrates such L-tyrosine, L-dihydroxyphenylalanine (L-DOPA), which also serve as bioregulators of melanogenesis [42,43]. Interestingly, the melanin precursors L-tyrosine and L-DOPA serve an additional role as hormonelike bioregulators, which is governed by melanocytes [44]. Darker skin contains more eumelanin than fair skin and is better protected against UV-induced skin damage. Furthermore, the epidermis of the darker skin allows only 7.4% of UV-B and 17.5% of UV-A to penetrate, in contrast to 24% UV-B and 55% UV-A that passes through fair (white) skin [45,46]. The capacity of several microbial strains to produce melanin could be relevant for skin health [47]. For instance, melanin harvested from cultures of *Streptomyces glaucescens* [48] exhibits anti-proliferative effects on human fibroblasts [23]. Melanin or melanin-like pigments can also be produced by fungi, but as their melanin production goes together with tissue invasion, their transplantation onto the skin might trigger an infection [49,50]. Fungi from the malassezia genus could be an exception, as some species can cause pityriasis versicolor, a condition with hyper- or hypopigmented skin patches, depending on the background color of the skin, while some others colonize the skin without symptoms [51,52]. Bacteria and fungi produce melanin as protection against UVR, solar or gamma radiation. For instance, melanization protects fungi such as *Cladosporium* spp. [53], *Sporothrix Schenckii* [54] and *Cryptococcus neoformans* [55] from UVR. With increasing evidence of UV-induced damage to skin microbiome, one could envision to use melanin as photoprotection not only for the skin, but also for the resident cutaneous microbes. *Escherichia* and *Enterococcus* species can produce serotonin, which is involved in skin pigmentation [56,57]. Indeed, inducing the production of melanin or transferring this capacity to non-melanogenic microbes using genetic modification has been discussed [58]. Interestingly, recombinant strains of *Bacillus thuringiensis* and *Pseudomonas putida*, which could produce increased levels of melanin, showed higher survival rate and resistance to UVR exposure [59,60]. Such (recombinant) microbes capable of producing and/or inducing melanin could be used for preventing the damaging effects of UVR on the resident microbiome and beyond. Apart from melanin, microorganisms can produce other compounds such as a large array of antioxidants [61]. *Malassezia furfur*, a human skin saprophyte, can produce pityriacitrin, a UV-absorbing indole alkaloid that protects the colonies from UVR [62]. The skin colonization with selected malassezia strains optimized for pityriacitrin production could have a clinical benefit in term of UV protection. On the other hand, toxins of *Malassezia furfur* may also lead to loss of pigment in the skin by complex mechanisms, including production of toxins such as dicarboxylic acids and inflammatory response [63,64]. Lastly, on the more classical probiotic front, the topical use of *Lactobacillus helveticus* supernatant on the skin has antioxidant effects on rodents [65]. Therefore, a novel approach to safely modulate pigmentation by microbes, or utilizing their antioxidation properties, could be beneficial for cutaneous photoprotection and preventing skin cancers (Figure 1). 

### 2.2. Protection from Photoaging

The skin consists of a complex network of cells and structures connecting cutaneous nerves, local neuroendocrine elements and the immune system [66,67]. UVR exposure accounts for 80% of the visible signs of skin aging, which includes dryness, scalping, wrinkling, disturbed pigmentation and solar freckles [67,68]. Multiple studies have shown that long-term exposure to UV-A leads to photoaging and free radical production, by damaging the dermis more significantly than UV-B [66,69]. The skin’s response to external stress such as UVR involves coordination between various local and systemic responses, which are mediated by the skin neuroendocrine system [70,71]. Photoaging is known to correlate with cancer risk, as for instance shown in a study from Central Europe where subjects with early signs of wrinkling on the neck were over four times more susceptible to melanoma compared to subjects from general population. Furthermore, subjects with skin freckling on the back also showed over three times the risk of developing melanoma (compared to control population) [68]. The risk of developing melanoma is known to correlate with intermittent UV exposure [68,72]. That said, the skin microbiome is known to change overtime, and is largely influenced by chronological and physiological skin aging [73]. Moreover, UVR is known to cause local (skin) [74] and systemic (gut) [36,75] changes in the microbial landscape. With increasing understanding of multidirectional interplay between gut microbiome and neuroendocrine system (microbiome-gut-brain axis) [76], it is tempting to speculate on its role in photoaging. 

Probiotics are emerging as potential therapeutic means to mitigate the damaging effects of UVR on the skin. Oral administration of *Bifidobacterium breve* in hairless mice prevented UVR-induced transepidermal water loss and suppressed UV-induced increase in hydrogen peroxide levels, oxidation of proteins and xanthine oxidase activity in the skin (Figure 1) [42]. Studies involving humans have also substantiated the role of probiotics in attenuating UV-induced skin damage. The probiotic *Lactobacillus johnsonii* was orally administered to UVR exposed-subjects and prevented the UVR-induced decrease in Langerhans cells and improved the recovery of immune homeostasis [77]. Other probiotics such as *Faecalibacterium prausnitzii* [45,46], *Lactobacillus plantarum* [47], *Bifidobacterium Breve Strain Yakult* [48], *Bifidobacterium longum* [47] and *Lactobacillus plantarum HY7714* [78] are known to have beneficial effects on photoaging in humans and mice. In depth reviews of probiotics in photoaging have been published elsewhere [72,79,80]. Moreover, orally administered prebiotics such as oligosaccharides are known to prevent transepidermal water loss, reduce erythema and prevent skin damage [81].

### 2.3. Anti-Tumor Effects of the Microbiome

UVR exposure has been associated with the development of different types of skin cancers. Chronic cumulative exposure is commonly associated with basal cell carcinoma (BCC) and squamous cell carcinoma (SCC). SCC can develop from actinic keratosis (AK), which is a typical lesion of photodamaged skin. Interestingly a recent study has shown higher relative abundances of commensal strains such as *Propionibacterium* and *Malassezia* in non-lesional skin compared to tissue of AK and SCC skin lesions. *Staphylococcus aureus* was significantly more abundant in lesional AK and SCC skin than non-lesional skin [53,54,55]. Moreover, *Staphylococcus aureus, Chlamydophila pneumoniae* and *Borrelia burgdorferi* have been associated with cutaneous T- or B-cell lymphoma [82,83]. Although studies on the role of skin microbiome in cancers is still in its infancy, initial reports, however, suggest an altered microbial landscape in cancerous skin that could be partly playing a role in disease pathogenesis and/or progression. Skin resident T cells play a role in the defense against formation and recurrence of skin neoplasia [84]. As the presence of bacteria on the skin surface can alter its T cell population [85], it is tempting to speculate that topical application of certain bacterial strains could act as secondary treatment against skin neoplasms. Skin exposure to UVR triggers inflammation [86] and studies suggests local (skin) and systemic (intestine) changes in microbial landscape after UVR exposure [36]. UVR-induced impairments of the immune system reduce the capacity of the host to reject skin cancers, and rather promote carcinogenesis. Moreover, microbial exposure plays a crucial role in cancer immunobiology by limiting chronic inflammation in early stages [87] and any disruptions of homeostatic skin microbiome could initiate inflammatory mechanisms that may lead to carcinogenesis. Overall, the response to UVR in the presence of microbiome on mice skin held in SPF conditions, compared to germ-free mice seem to be pro-inflammatory and protective [10]. Thus, the modification of certain strains could potentially be useful in fighting the development of cancerous cells locally. For instance, the colonization of mouse skin by strains of *S. epidermidis* that produce the antiproliferative agent 6-N-hydroxyaminopurine (6-HAP) diminishes the incidence of UV-induced skin neoplasms [88]. The systemic use of probiotics to control UV-B-induced immunosuppression has also gained interest, and oral intake of lipoteichoic acid from *Lactobacillus rhamnosus* decreased the number of UV-induced skin tumor in SKH-1 hairless mice [89]. Prebiotics, capable of inducing or limiting the growth of certain microbes, are now considered to modulate the growth of certain pathogenic skin microbes. Oral intake of prebiotics such as inulin and mucin have been reported to induce *Bifidobacterium* spp. [90] and *Akkermansia muciniphila* [91,92], which are involved in inhibiting melanoma growth [93]. Addition of inulin and mucin to the diet induces anti-tumor immune responses and inhibits subcutaneously implanted BRAF mutant melanoma in a syngeneic mouse model [94]. There is a growing body of evidence that microbiome enhances the effectiveness of chemotherapy, radiotherapy and immunotherapy [95,96]. However, the interplay between photo(chemo)therapy, used to treat various inflammatory diseases such as psoriasis, atopic eczema, lichen planus and graft-versus-host disease (GvHD) as well as neoplastic diseases such as cutaneous T cell lymphoma and other immunoproliferative conditions, and the local microbial environment needs to be addressed. Finally, recent reports have shown that antibiotic treatment or selective enrichment of certain microbial species in the gut can indeed limit the effectiveness of immune check point therapy in cutaneous melanoma [97,98]. Interestingly, a recent study demonstrated targeted depletion of bacteria from a mixed population by programmed inhibitor cells that can direct the antibacterial activity against specified target cells using the type VI secretion system (T6SS) [99]. Such an approach where topical treatment selectively enriches skin commensals or targets specified microbes (pathogenic), known to be involved in immune responses [100], could also be envisioned for modulating UVR-induced immune suppression and the efficacy of phototherapy (Figure 1).

### 2.4. Enhancement of the UVR-induced Immune Suppression

UVR has a profound effect on the skin’s immune system, which can manifest both locally and systemically in a dose-dependent manner. The role of UVR in modulating adaptive immunity was shown in landmark studies by Kripke et al., where the mice exposed to sub-carcinogenic doses of UVR developed skin tumors due to further suppression of the anti-tumor immune response [101]. Several subsequent studies in mice and humans established that UVR-induced immune suppression was a result of activation of suppressor T cells (now called as regulatory T cells (Tregs)), modulation in the number and function of antigen presenting cells, activation and recruitment of neutrophils and increased expression of anti-inflammatory cytokines such as IL-4 and IL-10 [37]. The role of skin microbiome in UVR-induced immune suppression has also now been addressed. We have recently shown, using the contact hypersensitivity (CHS) model, that absence of microbiome enhanced UVR-induced immune suppression in mice lacking microbiome (germ-free), with predominant expression of anti-inflammatory cytokines such as IL-10, and increased numbers of monocytes/macrophages in the skin. In contrast, mice with a microbiome showed diminished UVR-induced immune suppression with higher expression of pro-inflammatory cytokines such as IL-1β, epidermal hyperplasia and neutrophilic infiltration [10]. Interestingly, administration of fermented milk containing *Lactobacillus casei* DN-114 001 in a CHS model reduced skin inflammation by inhibiting the hapten-specific CD8+ T cells [102], and enhancing the frequency of FoxP3+ regulatory T cells and IL-10 producing CD4+CD25+ effector T cells [103]. These studies in T cell-mediated allergic skin inflammation model demonstrate that certain microbes or pro-biotics can indeed reduce severity of the disease by regulating the immune response (Figure 1). Other skin commensals could show similar effects, which remains to be addressed.

### 2.5. Protection in UVR-induced Skin Inflammation

Several skin diseases are triggered by UVR, such as cutaneous lupus [104], or polymorphic light eruption (PLE) [105]. In cutaneous lupus, UVR exposure induces long-lasting molecular changes in keratinocytes, including increased inducible nitric oxide (NO) synthase (iNOS) and interferon-stimulated transcripts [106]. For these diseases, the use of topical anti-inflammatory probiotics could be beneficial. For instance, *Lactobacillus reuteri* showed anti-inflammatory properties on a reconstructed human epidermis upon UVR-induced inflammation [107]. Oral intake of fermented milk, exopolysaccharide or *Lactobacillus planterum* in mice reduced UVR-induced epidermal thickness and activity of metalloprotease [108]. That said, oral administration of probiotic supplements containing lycopene, beta-carotene and *Lactobacillus johnsonii* [109] or natural extracts from fern leaves, such as *Polypodium leucotomos*, has been previously described to prevent and/or diminish PLE symptoms [110,111]. Patients with PLE have been reported to have low levels of vitamin D, due to sun avoidance. UVR-induced vitamin D and its metabolites participate in various immune pathways [112], leading to immune suppression by influencing innate and adaptive immune cells [113]. High doses of vitamin D_3_ can also alter the microbial composition in the gut [114]. Recently, it has been shown that exposure to narrow band UV-B increases serum vitamin D levels, which then modulates intestinal microbiome [36]. In this context, PLE pathogenesis, which is (hypothetically) linked with altered skin microbiome or microbial elements, could be treated/prevented using vitamin D supplements [105]. A clinical study has in fact shown that topical pre-treatment with a 1,25-dihydroxyvitamin D_3_ analogue (calcipotriol) significantly reduced photo-provoked PLE symptoms [115]. Though the mechanism of this has remained elusive, this finding might be partly due to microbial modulation by calcipotriol and/or its metabolites. Moreover, UVR-induced vitamin D3 can help “program” the migration of T cells within the skin [25]. 

### 2.6. Controlling Inflammatory Diseases in UV-exposed Skin Areas

Acne, rosacea and seborrheic dermatitis are an interesting group of mostly facial diseases that occur in UVR-exposed skin, but in which no direct effect of UVR is proven in the pathophysiology. It is not known whether the UVR effects on skin possibly participate in an inappropriate immune response to local microbes. UV-B triggers inflammasome activation, which is potentialized by the presence of the antimicrobial peptide LL-37 [116]. LL-37 is produced by the skin following the exposure to *P. acnes* and *Malassezia* [117], and is induced by vitamin D in sebocytes [118], which could be a mechanism through which saprophytes might become proinflammatory on UV-exposed areas. In this context, microbes with anti-inflammatory effect could be of interest. In mice, the application of the short chain fatty acid (succinic acid) produced by *S. epidermidis* onto ears injected with *P. acnes* decreases local inflammation [119]. In humans, the scarcity of randomized controlled trials of pre- and probiotics in facial dermatosis makes it difficult to draw any conclusions. Among the few randomized trials, the local application of *Vitreoscilla filiformis* decreased the clinical score in seborrheic dermatitis in one trial [120]. In mice, this bacterium impacted dendritic cells and induced IL-10+ regulatory T cells and reduced AD-like inflammation [121]. In many other skin diseases, such as psoriasis and eczema, the exposure to natural light or to UVA/B is, however, used as a treatment. For these diseases, the question is merely if the use of anti-inflammatory pre- or probiotics could potentiate the benefit [122]. Hence, in the last decade, elegant mice data have shown that the skin microbiome has been able to modify local T cell population and poise its function [85,123]. As T cells can dwell and persist in the skin after infections [124,125], using anti-inflammatory bacteria to maintain the balance of local pool of cutaneous immune cells will hopefully open interesting perspectives for better protection against UVR (Figure 1). Moreover, skin microbiome transplantation (live-biotherapeutic approach) has been successfully demonstrated to reduce atopic dermatitis (AD) symptoms. Culturable gram-negative bacteria (*Roseomonas mucosa*) collected from healthy humans were associated with activating the innate immune system, enhanced the barrier function and controlled the growth of *S. aureus* in AD-like mouse model (MC903) [126]. This approach of topical application of live-biotherapeutics also successfully reduced AD disease severity and *S. aureus* burden in AD patients [127]. A similar approach of using live-biotherapeutics should be envisioned to control UV-induced inflammatory diseases. 

## 3. Conclusions and Outlook

Overall, probiotics and prebiotics are promising in protecting the skin against UVR-induced skin damage. On the other side of the spectrum, pro- and prebiotics may also modulate the efficacy of phototherapy. With growing knowledge of the human microbiome and patients’ unique microbial “fingerprint,” personalized oral or skin care regimen should be envisioned using special formulations to promote commensal microbiome that can protects against UVR and suppress growth of pathogens. Using live microbes pose certain side effects such as infection, deleterious metabolic activity, excessive immune stimulation, and dysregulation in gene expression in susceptible individuals [128,129,130]. On the prebiotic front, although they are well tolerated, undesirable effects after oral administration may occur, with allergic reactions and abdominal discomfort, and even potential distant effects on the skin [129,131]. To succeed in using probiotics and prebiotics, many questions need to be answered: what is the best route of administration of a supplementation? Will a combination approach of pro- or prebiotic supplementation be most effective? Are live microbes more advantageous than using pro- or pre-biotics, and how do we successfully implant them without side-effects? To address these issues, further studies are warranted that will enhance our understanding of the potential of skin microbiome, pro- and pre-biotics and help in developing novel strategies to control the cutaneous interplay with UVR.
